# A Systematic Review of the Epidemiology of Echinococcosis in Domestic and Wild Animals

**DOI:** 10.1371/journal.pntd.0002249

**Published:** 2013-06-06

**Authors:** Belen Otero-Abad, Paul R. Torgerson

**Affiliations:** Vetsuisse-Faculty, University of Zurich, Section for Veterinary Epidemiology, Zurich, Switzerland; Universidad Peruana Cayetano Heredia, Peru

## Abstract

**Background:**

Human echinococcosis is a neglected zoonosis caused by parasites of the genus *Echinococcus.* The most frequent clinical forms of echinococcosis, cystic echinococcosis (CE) and alveolar echinococcosis (AE), are responsible for a substantial health and economic burden, particularly to low-income societies. Quantitative epidemiology can provide important information to improve the understanding of parasite transmission and hence is an important part of efforts to control this disease. The purpose of this review is to give an insight on factors associated with echinococcosis in animal hosts by summarising significant results reported from epidemiological studies identified through a systematic search.

**Methodology and Principal Findings:**

The systematic search was conducted mainly in electronic databases but a few additional records were obtained from other sources. Retrieved entries were examined in order to identify available peer-reviewed epidemiological studies that found significant risk factors for infection using associative statistical methods. One hundred studies met the eligibility criteria and were suitable for data extraction. Epidemiological factors associated with increased risk of *E. granulosus* infection in dogs included feeding with raw viscera, possibility of scavenging dead animals, lack of anthelmintic treatment and owners' poor health education and indicators of poverty. Key factors associated with *E. granulosus* infection in intermediate hosts were related to the hosts' age and the intensity of environmental contamination with parasite eggs. *E. multilocularis* transmission dynamics in animal hosts depended on the interaction of several ecological factors, such as hosts' population densities, host-prey interactions, landscape characteristics, climate conditions and human-related activities.

**Conclusions/Significance:**

Results derived from epidemiological studies provide a better understanding of the behavioural, biological and ecological factors involved in the transmission of this parasite and hence can aid in the design of more effective control strategies.

## Author Summary

Echinococcosis is considered a neglected zoonotic disease caused by the larval form of *Echinococcus spp*. tapeworms. Humans become infected through the accidental intake of parasitic eggs excreted by the faeces of definitive hosts (dogs, foxes and other canids). Infection involves the development of cysts, primarily in the lungs and liver, causing damage as they enlarge like a slowly growing tumor. Transmission is facilitated by the general lack of awareness of infection factors and epidemiological models can identify them. Nevertheless, there has never been a systematic review summarizing the significant determinants for echinococcosis in animals. One hundred publications were included in the results after evaluating 1,935 entries and screening the references lists of the eligible papers. Principal factors associated with canine infection included the access of dogs to infected offal, allowing dogs to roam free, being a young and/or male dog and social behaviours linked with poor health conditions and poor living environments of dog owners. Ecological factors influencing *E. multilocularis* transmission encompassed population densities of foxes and rodents, predator-prey relationships, geographical characteristics, climate conditions and the movement of foxes towards urban areas. These findings are important, as intervention to control echinococcosis requires intervention in animal populations.

## Introduction

Echinococcosis is a zoonotic parasitic infection caused by the larval stage of several species belonging to the genus *Echinococcus*. Human echinococcosis results following the direct or indirect infection from canid hosts, which are themselves infected by various domestic and wild mammals. *Echinococcus spp*. are found throughout the world, although some species have restrictive distributions. Echinococcosis is a major public health concern, particularly in developing regions with limited economic resources. Furthermore, there are indications of an increasing number of cases in certain areas so it is becoming an emerging or re-emerging disease [1]–[4].

This article will focus on *E. granulosus* and *E. multilocularis*, as these are responsible for virtually all the human and animal burden of the disease. *E. granulosus* is now recognised as having a number of genotypes and molecular evidence suggests there may be a number of species. Hence, *E. granulosus* genotypes 1–10 are now commonly referred to as *E. granulosus sensu stricto* (genotypes G1–G3), *E. equinus* (G4), *E. ortleppi* (G5) and *E. canadensis* (G6–G10) [5]–[7]. Additionally, mitochondrial studies have identified *E. felidis* as a distinct species although phylogenetically closely related with *E. granulosus sensu stricto* [8]. Of these, *E. granulosus* sensu stricto, *E. ortleppi* and *E. canadensis* cause human cystic echinococcosis (CE) whilst *E. multilocularis* causes alveolar echinococcosis *(AE). E. equinus* is not believed to be zoonotic and the pathogenicity of *E. felidis* to man is unknown.

CE is usually maintained by the domestic cycle (dog/domestic ungulate) and represents a persistent zoonosis in rural livestock-raising areas where humans cohabit with dogs fed on raw livestock offal [9]. AE is mainly supported by a sylvatic cycle (fox/rodents), which can be linked with domestic dogs and cats [10]. AE is confined to the northern hemisphere, representing a major endemic disease in the western and northwestern parts of China [11]. High infection rates have also been reported for domestic dogs in China [12], [13], where they are likely to play a significant role in human infection [14], [15]. It is also an emerging disease in central Europe coinciding with the growth of fox populations and their expansion towards the urban areas [1]. Although AE is less common than CE it poses a major threat to human health since it is more difficult and costly to treat.

Echinococcosis infection constitutes a significant financial constraint derived from human health costs and livestock production losses. The global burden of CE and AE has been calculated to be of approximately 1 million and 600,000 DALYs respectively [16], [17]. In addition the economic burden of CE on the global livestock industry has been estimated at over $2 billion per annum [16]. Despite the substantial socioeconomic impact, CE and AE remain neglected zoonoses [18].

A sound understanding of the epidemiology of infection in animals is a key factor in limiting the transmission to humans. Controlling the parasitic infection in animals is crucial to reduce the incidence of human disease. The study of *Echinococcus* transmission on animal hosts draws heavily on statistical and epidemiological models. Modelling enhances our epidemiological understanding of parasite transmission allowing predictions to be made and thus, the evaluation of potential control strategies in a cost-effective way. Moreover, the World Health Organization has recently included human echinococcosis within the group of neglected tropical diseases, and recommends a veterinary public health strategy as part of an effective control approach [19]. However, to the authors' knowledge, a study summarizing risk factors that have been found to have significant association with *Echinococcus* infection in animals is lacking. The purpose of this review is to provide an exhaustive summary of determinants that were found to be significantly associated with *Echinococcus* infection in animal hosts, in order to better understand the parasite epidemiology. This knowledge will assist in the design of effective control programmes to reduce transmission to humans.

## Materials and Methods

The objective of this study is to review the current state of understanding on risk factors for echinococcosis in animals by presenting significant results from epidemiological associative studies collected in a systematic way. Associative studies determine the strength of association between disease occurrence and suggested risk factors. These studies employ a number of commonly used statistical techniques defined in Table S1 (Table S1).

Principal data sources selected to carry out the literature search included six bibliographic databases: PubMed, Scopus, Web of Knowledge, Cab Direct, Science Direct and Google Scholar. The computer search was not constrained by language or date, although the eligibility criteria were restricted to 5 languages. The online search was conducted by combining topic-related keywords using Boolean operators. The asterisk (*), when used, expanded the search by looking for words with similar prefixes (i.e. echinococc* will search for echinococcus, echinococci, echinococcosis, echinococcoses). Different combinations were tailored for each electronic database in order to narrow the amount of results retrieved but at the same time maximizing the number of relevant studies. The last online search was performed on the 15th October 2012. Table 1 illustrates the number of papers identified in each database.

**Table 1:** Search strategies and results for 6 electronic databases^1^.

Database	Search strategy	Results
PubMed	“echinococcus”[Mesh Terms] AND “epidemiologic factors”[MeSH Terms]) AND “animals”[MeSH Terms]	130
Scopus	TITLE-ABS-KEY (echinococcus AND epidemiolog* OR factor* AND dog* OR fox* OR livestock) AND SUBJAREA (mult OR medi OR vete OR heal)	466
Web of Knowledge	Topic = (echinococcus) AND Topic = (epidemiolog* factor*) AND Topic = (animal*)	302
Cab Direct	(echinococc*) AND (epidemiolog*) OR (factor*) AND (dog*) OR (fox*) OR (animal*)	366
Science Direct	(echinococc*) AND (epidemiolog* factor*) AND (animal*) AND LIMIT TO (topics, “echinococcus granulosus, echinococcus multilocularis, veterinary parasitology, cystic echinococcosis, hydatid disease, tropical medicine, alveolar echinococcosis, hydatid cyst, Infectious disease, parasitic zoonosis, red fox”)	301
Google Scholar (1)	TITLE-(Echinococcus multilocularis foxes)	130
Google Scholar (2)	TITLE-(Echinococcus granulosus dogs)	240

1 Last search performed on the 15^th^ October 2012.

At the first selection stage, the titles and/or abstracts of the studies retrieved were screened for relevance to the topic. At the second stage, the full texts of retained documents were examined to detect eligible studies. The eligibility criteria encompassed available publications in certain languages (English, Spanish, Italian, French and German), type of study (peer-reviewed epidemiological analytical studies), methodology applied (associative statistical methods) and results (statistically significant findings). Remaining records were combined to eliminate duplicate publications. Furthermore, the reference lists of the selected studies were examined as a method to supplement the electronic searching.

Data were extracted from the selected studies by filling tables containing the four following sections: article reference, study information, statistical method applied and significant factor/s reported. Data on study characteristics included: study description, geographic location, type of animal host studied, sample size and statistical analyses performed. If the analysis was undertaken with multiple explanatory variables, only variables that remained significant were included. Disease determinants were reported along with their significant p-values (*p*<0.05) or equivalent measure of goodness of fit, such as the Akaike information criterion (AIC), the coefficient of determination (*R^2^*) or 95% confidence intervals, retrieved from tables and text of primary articles. Furthermore, measures of association between significant risk factors and infection are also reported when available (e.g. Odds ratio).

The systematic review followed PRISMA guidelines and a PRISMA check list is provided as supplementary material (Checklist S1).

## Results

**Figure 1:**
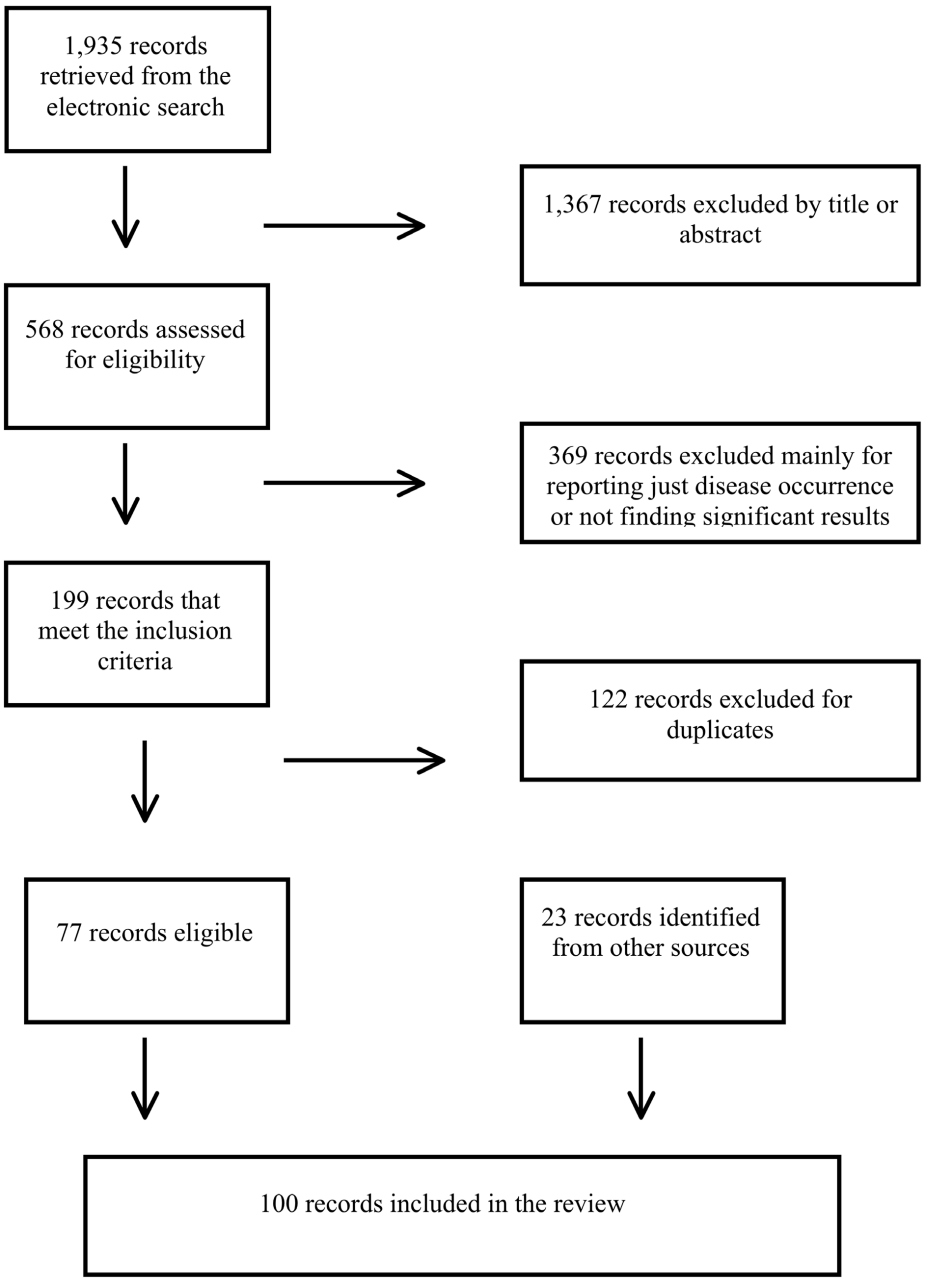
Literature search flow diagram.

The literature search yielded 1,935 potentially relevant references (see Table 1). After the first screening by title and/or abstract, 568 remaining publications were assessed by a full text examination. Of the 369 articles discarded during this second selection, the two most common reasons for exclusion were if only measures of disease occurrence (prevalence) were reported and if there were a lack of statistically significant factors. Other reasons for exclusion included language, presenting non-original results, article availability or when the statistical method used for the analyses was not associative. A total of 100 references were presented in the review tables, including 23 additional articles retrieved from the screening of references lists of the eligible papers. The flow diagram in Figure 1 shows the review process.

This review presents some limitations with regards to missing publications, language bias and publication bias. The combination of terms entered in each individual computer search aimed to retrieve as many relevant publications as possible but at the same time tried to narrow the amount of results. Hence, it is highly possible that relevant papers, which did not contain in their titles or abstracts the key words used in our search, may have been overlooked. In addition, just around 5% of the articles selected were not written in English, indicating a major bias towards English publications. Furthermore, about 95% of selected papers were obtained through electronic search. Thus, a bias towards articles published online has to be acknowledged. Additionally, this review has a strong bias towards articles reporting positive findings. Nevertheless, it was decided from the beginning that significant findings were a requirement for eligibility of inclusion. Finally, it is worth remembering that, in research, significant results are the ones reporting p-values less than 0.05. Yet, this is just an agreed threshold to have a convenient and standardised way to assess the statistical significance of an effect.

In addition, the majority of the studies included in this review were cross-sectional studies reporting *Echinococcus* infection and associated risk factors at a specific point in time. These types of studies can be subjected to selection and information bias. Common sources of potential bias affecting *E. granulosus* studies can be borne from recall errors or non-responded questionnaires from dog owners, non-randomly selected animals (e.g. abattoir studies) or misclassification bias due to imperfect sensitivity and specificity of the diagnostic test used (e.g. aerocoline purgation or coproantigen ELISA). Common sources of potential bias in *E. multilocularis* studies included the selection of sampled animals being based just on availability (e.g. foxes shot or found dead) and misclassification when the diagnostic test used was other than necropsy. Although acknowledging potential bias, no studies were excluded for qualitative reasons.

### Associative models for *E. granulosus* in definitive hosts

#### Dogs

The predominant life cycle of *E. granulosus* takes place in a synanthropic cycle with domestic dogs (*Canis lupus familiaris*) as definitive hosts and livestock animals as intermediate hosts. A number of factors have been found to influence the frequency and intensity of canine echinococcosis. The most important of these is the potential access that dogs have to uncooked and infected offal. The determinants that might increase access to offal include food sources, access to the location where animals are slaughtered, access to livestock rearing areas and carcasses, non-urban location of dogs, whether dogs are free to roam, the type of dog, the knowledge of the owners about echinococcosis and their socioeconomic background. Other determinants of canine echinococcosis include the age and gender of the dogs, and if the dogs receive anthelmintic treatment.

The feeding of domestic dogs with infected offal perpetuates *Echinococcus* transmission (Table S2). Dogs known to eat raw offal or infected viscera were reported more likely to be coproantigen positive for *E. granulosus*. [20], [21]. Similarly, activities that prevent the consumption of livestock offal by dogs, such as the proper disposal of animal carcasses by incineration/burial or not performing home slaughtering, were found protective factors for dogs' infection [21], [22].

Likewise, dogs with more possibilities to have contact with livestock were more likely to become infected. Dogs from a semi-nomadic pastoral community in north-west China presented more than 2.5 times higher coproantigen positivity in the winter area than in summer pastures [23], possibly due to greater availability of offal when animals are slaughtered. Farm dogs and sheepdogs showed higher infection rates than other type of dogs [20], [24], [25]. In Patagonia, Argentina, a positive correlation between livestock premises showing higher canine coproantigen positivity and their number of reared sheep was found [26]. Similarly, dogs living in rural communities, or with access to fields, presented a higher risk of infection compared with urban dogs [22], [24], [25], [27], [28]. Nevertheless, a study reported lower odds of a dog being copropositive in rural sites and towns compared to cities, although the same study found higher prevalence in dogs from urban households located in the periphery of a city, near to rural areas [22]. In Tunisia dogs located within 1 km of a refuse dump presented high infection rates [29].

The ability of dogs to roam freely was one of the most commonly reported risk factors for *E. granulosus* infection. Several studies reported that dogs which were free to roam presented an increased risk of being coproantigen positive, compared to indoor or chained dogs that were restrained most of the time [21], [27], [30]–[33]. Likewise, stray dogs showed greater intensity of infection compared with domesticated dogs [34].

Several studies reported a higher risk of *E. granulosus* infection in young dogs compared to adults (Table S3). Higher canine prevalence was commonly reported in young animals (<2 years) [22], [35]. Likewise, dogs older than 5 years showed lower coproantigen positivity, and even lower parasite burden, compared to younger groups [21], [24], [34].

Although numerous studies recorded higher prevalences in males compared to females, just one study was found to report this difference as significant [27].

Seven retrieved studies supported the existence of an increasing risk for canine infection and some socio-economic factors associated with dog ownership (Table S4). Risk factors for *E. granulosus* infection were associated with the dog owner's lack of knowledge about parasite transmission or deficiencies in the anthelmintic treatment [22], [24], [27], [31], [33]. Additionally, the cultural and economic background of the owners was found to be related to infection risk in dogs. In Cyprus, the percentage of Turkish Cypriots in the village explained, approximately 9% of the total variance in the prevalence of canine echinococcosis [36]. Likewise, the Maori population represented a major obstacle for the success of an echinococcosis campaign in dogs in New Zealand [37].

### Associative models for *E. granulosus* in intermediate hosts

#### Livestock

The transmission cycle of *E. granulosus* relies primarily on the domestic cycle where farm species act as intermediate hosts. Principal determinants of livestock infection found in the literature encompassed the level of environmental contamination with parasite eggs and age of the host, among others (Table S5).

Significant differences in prevalence of cystic echinococcosis between study locations or different livestock origin have been repeatedly reported [38]–[45]. Seasonal variations in hydatidosis prevalence were also recorded through abattoir meat inspection [46], [47]. Other environmental factors found associated with CE in livestock were high altitudes and increasing annual rainfall [44], [48].

The age of the host has been largely recognised as an infection determinant for many farm species. Numerous studies have recorded higher hydatidosis prevalence in old animals compared to young ones [41], [43], [49]–[56]. Small ruminants (sheep and goats) equal or older than 3 years old were also found to be 1.6 times more at risk compared to the younger groups [57]. Additionally, an increase of cyst abundance has been reported in older age groups of farm animals [47], [55], [58], [59].

The gender of the intermediate host has also been identified as a possible determinant of CE, although reports were inconsistent. In a large slaughterhouse survey in Saudi Arabia, females were found significantly more likely to be infected than males for cattle (OR 1.76; 95%CI 1.27, 2.43) and sheep (OR 1.21; CI 1.01, 1.44) [47]. Females were also reported showing higher prevalence than males in eastern Libya [54], Kuwait [60], Iran [61] and in China [62]. Contrarily, a study carried out in Ethiopia revealed that small male ruminants were significantly more susceptible to infection compared to the females [51].

Significant differences in CE prevalence were consistently found among host species. However, reported studies differ on which farm species presented the highest rates. Small ruminants have frequently been observed showing high rates of infection [47], [63], with sheep registering higher risk of infection compared to goats [51], [54], [57]. Cattle have also been identified in many studies as bearing the highest prevalence of CE of those observed in farm species [40], [44], [48], [64]–[66]. A study reported camels as the domestic intermediate host most likely to be infected, although cattle were recorded with the highest cyst intensity [47].

Finally, farm location and management factors were reported to be associated with hydatid disease in livestock. Local cattle breeds showed higher cyst prevalence than crossbreeds in an Ethiopian study [67]. Pigs reared in intensive conditions reported significantly lower prevalence compared to pigs reared in free-range conditions or on family farms [50], [68]. While sheep and goats from mixed farming systems showed higher rates of hydatid infection compared to small ruminants from pastoral systems [51]. In a geo-referenced study carried out on cattle and water buffalo farms, showed that the distance from positive testing cattle farms to sheep farms were significantly lower than for positive testing water buffalo farms. Cattle had higher prevalences (20.0%, 95%CI 18.5–21.6%) than water buffaloes (12.4%, 95%CI 10.0–15.4%) [64].

#### Wild intermediate hosts

CE has been recorded in a large number of wild animals, even although wildlife studies rarely report more than point prevalence estimates. A publication was found to report that kangaroo females were twice as likely to be infected as males [69]. Other studies reported that there was an increasing prevalence and intensity of cysts in correlation with an increase in the density, and age, of the moose population [70], [71] (Table S6).

### Associative models for *E. multilocularis* in definitive hosts

#### Foxes

In contrast with the domestic cycle of *E. granulosus*, the transmission of *E. multilocularis* is primarily supported by foxes and small mammals [72]. Although the Red fox (*Vulpes vulpes*) has been identified to be the most common definitive host, other fox species such as the Arctic fox (*Vulpes lagopus*, formerly *Alopex lagopus*), the Corsac fox (*Vulpes corsac*) or the Tibetan fox (*Vulpes ferrilata*), are also susceptible to infection [73].

Factors identified in this review as contributing to the infection rates of *E. multilocularis* in foxes include; host population dynamics, interactions with prey animals, spatial distribution, seasonal changes and age. As such factors are interrelated it can be challenging to resolve independent risk factors for infection.

There is extensive literature linking young foxes with *E. multilocularis* infection (Table S7). Many epidemiological studies have reported a higher prevalence and/or abundance in juvenile foxes (<1 year old) compared with adults [74]–[80]. However, some researchers have found that this relation between parasite infection and host age is influenced by other factors. In Germany, under high-endemic conditions young foxes were found to be more frequently infected than adults whereas in low-endemic areas infection rates were higher in adults (OR 2.25, 95%CI 1.26–4.02) [81]. In Switzerland, seasonal changes of prevalence were found to be more pronounced in juveniles than in adults (i.e. summer/autumn×juvenile vs. winter×adult (OR 0.36, 95%CI 0.14–0.91). Whereas prevalence differences that related to the type of urbanization level were more pronounced in adults (i.e. urban×juvenile vs. peri-urban×adult (OR 4.76, 95%CI 1.26–17.39) [82].

There is less scientific evidence to support that being a male or female fox act as an independent variable influencing the infection status of the animal. Just one study identified being a male as a significant regressor parameter associated with the mean parasite abundance in foxes [83].

Environmental factors seemed to play a critical role in *E. multilocularis* infection in foxes (Table S8), resulting in a heterogeneous geographical distribution of the parasite [81], [84]–[86].

Specific geographic-related features can act directly upon parasite transmission. For example, in Germany significant differences in prevalence were reported between 3 different locations (i.e. Zone1 vs. Zone2, OR 2.64, 95%CI 1.92–3.64 or Zone1 vs. Zone3, OR 4.9, 95%CI 3.12–7.73) [81]. In the same country, the highest parasite burdens were found in foxes from regions with a high quota of agricultural land and precipitation [87]. In France, mid-altitude areas with a high proportion of permanent grassland showed higher fox prevalence when compared with low altitude sampling locations [88]. Likewise, regional meteorological conditions, such as low temperatures or high annual precipitation, have been reported as being associated with the infection rates in foxes. For instance, a significant correlation was established in Slovakia between *E. multilocularis* prevalence/abundance and the increasing mean annual rainfall [89], [90]. Inversely, a negative association between the infection of foxes and annual temperature was recorded in the German Saxony [91].

Similarly, infection rates in foxes have been documented to vary between seasons [92], [93]. In Belgium, foxes collected in summer and autumn were more often infected than the ones collected in winter and in spring [93]. Sometimes these seasonal variations in prevalence were found to also relate to other factors. In Zurich, Switzerland, seasonal changes of prevalence were observed to be more pronounced in juveniles (<1 year old) than in adult foxes (i.e. Summer/autumn×juvenile vs. winter×adult, OR 0.36, 95%CI 0.14–0.91) [82]. Again in Zurich, significant seasonal differences could only be established in sub-adult male foxes caught within the urban area [76]. Variations in prevalence between seasons and geographic location were also found to be dependent on host age in western Switzerland [75].

As previously mentioned, the spatial distribution of *E. multilocularis* in foxes was found to be linked to regional geographic and climatic conditions (Table S9). Several spatial studies have identified disease clusters or locations where foxes presented higher parasite prevalence [91], [94]–[96]. Spatial studies on *E. multilocularis* in foxes have also helped to establish associations between location of infection, landscape characteristics and ecological factors. In France, the percentage of grassland was associated with fox coproantigen distribution [97]. In Germany infected foxes were more frequently caught near humid areas and pastures [98]. Whereas, in Svalbard (Norway), positive infected faeces from the artic fox were confined within the habitat of the only intermediate host available, the sibling vole (*Microtus levis*) [99].

Transmission dynamics of *E. multilocularis* depend directly on the densities and predator-prey relationship between definitive and intermediate hosts. These two factors differ greatly among the level of urbanization in different areas (Table S10). Despite a higher prevalence in foxes from rural areas when compared with urban areas [100], there is a high infection pressure frequently reported in the periphery of the cities [78], [101]. Some studies found that the association between infection status and type of urbanization zone was related to other variables such season or age of the host. In Zurich, higher infection rates during winter were recorded in rural and peri-urban foxes compared with urban animals [76], [102]. In the same city, prevalence variations between urban types were more pronounced in adults than juveniles (i.e. Spring×juvenile vs. peri-urban×adult, OR 0.23, 95%CI 0.06–0.89) [82].

Many authors have highlighted the importance of the availability and predation level on potential intermediate hosts for the successful transmission of *E. multilocularis*. The relationship between parasite prevalence in foxes and vole abundance was reported in Hokkaido (Japan), where infection rates in foxes were proved to be dependent upon the current-year abundance of voles [103]. Likewise, several publications have evidenced a significant correlation between parasite prevalence in foxes and the density [89], prevalence [93] and predation of potential intermediate host populations [104]. Additionally, the infection level in foxes is also dependant on fox population density [105].

#### Other carnivores

Some wild carnivores, members of the family Canidae and Felidae, can harbour *E. multilocularis*. Disease determinants for *E. multilocularis* infection in definitive hosts, other than foxes, appeared to be associated with greater exposure to infected intermediate hosts (Table S11). As in foxes, canine infection was linked with the abundance and availability of potential intermediate hosts [106], [107]. Dogs that preyed on rodents were more likely to be infected [108]. Similarly, non-restrained dogs or hunting dogs were identified as having greater exposure to rodents, and thus, to infection [12], [109]. In Germany, regional differences in canine prevalence were observed between the north and the south [110]. Other carnivores, such as racoon dogs, showed seasonal variations in prevalence [83] whereas higher prevalence was recorded in young (<1 year old) [111] and male coyotes [112].

### Associative models for *E. multilocularis* in intermediate hosts

#### Voles

More than 40 species of small mammals (rodents and lagomorphs) can act as intermediate hosts for *E. multilocularis* [10]. Among them, grassland rodents (i.e. *Arvicola terrestris* or *Microtus sp.*) have been identified as playing an important contribution to the diet of foxes and on cestodes transmission [113].

The risk of *E. multilocularis* infection in rodents is influenced by ecological and environmental factors that ultimately shape their numbers and age-structure (Table S12). Voles' annual population fluctuations had a significant effect on the yearly prevalence recorded in *A. terrestris* [114]. Environmental factors such as type of habitat or climatic season and their derived interaction terms, were found to explain much of the variance observed in parasite prevalence in the deer mouse (*Peromyscus maniculatus*) [115]. Low average day temperatures significantly increased the infection risk in *A. terrestris* [116]. Geographic location and sampling site have also been reported to be associated with infection rates in voles [102], [116]–[118]. Prevalence of *E. multilocularis* in rodents has been frequently associated with their increasing length and body size, which is linked to maturity and age [117]–[119]. Adult voles have frequently shown higher prevalence compared to sub-adults or juveniles [93], [102], [116].

Table 2 presents the summary of key findings reported in this review.

**Table 2:** Key findings.

Causative agent	Host	Risk Factors
*E. granulosus*	Dog (definitive host)	- Feeding with raw viscera, being a farm, rural or stray dog or being untied or free to roam
		- Being a young and/or male dog
		- Dog owner's lack of knowledge about hydatid disease and the lack of deworming treatment in dogs plus the owners' ethnic origin (linked with poor health education and deprived living conditions)
*E. granulosus*	Domestic livestock (intermediate hosts)	- Increasing hosts' age, geographical location, meteorological conditions, female gender, host species and type of farming management
*E. granulosus*	Wild life (intermediate hosts)	- Hosts' age, female gender and hosts' densities
*E. multilocularis*	Fox (definitive host)	- Being a young and/or male fox
		- Climatic conditions and geographic location (marked spatial distribution)
		- Host population dynamics and interactions with intermediate hosts (rodents), frequently influenced by urbanization level
*E. multilocularis*	Other canids (definitive host)	- Feeding with raw viscera, being hunting dogs or free to roam and availability of rodents
*E. multilocularis*	Rodents (intermediate hosts)	- Increasing adult age
		- Meteorological and geographical conditions
		- Rodent's densities

## Discussion

Human echinococcosis is a widely distributed parasitic infection, which despite adding a significant health and economic burden to the human race, is still a neglected disease [120]. A sound understanding of the epidemiology of *Echinococcus* in animal hosts is essential for designing an effective control programme [18]. To the authors' knowledge, this is the first study to systematically collect data on the infection determinants of *Echinococcus* in animals.

CE is a widespread chronic zoonosis, and domestic dogs have long been identified as the main infection source for humans. Dogs acquire *E. granulosus* through the ingestion of viscera from infected intermediate hosts. Factors facilitating the contact of dogs with raw offal are potential determinants for canine infection. Dogs from a semi-nomadic pastoral community showed higher infection levels in winter when higher numbers of livestock are slaughtered for the winter provisions [23]. Being a farming dog has been established as a risk factor for *E. granulosus* infection since they usually have higher contact with livestock, which can be seen as a proxy for scavenging on infected carcasses [20], [24], [25]. Hence, the risk of *E. granulosus* infection in dogs is commonly higher in rural areas [28]. However, high infection rates have also been recorded in dogs from the borders of urban areas. The continuation of the practice of home slaughtering in the periphery of some cities might explain these findings [22]. Similarly, dogs allowed to roam [27], [30]–[32] or stray dogs [29], [34] have also been identified as presenting higher infection risk as they have increased possibilities of finding and ingesting raw carcass meat and offal of fallen livestock. In contrast, dogs that cannot roam freely, like guard-dogs or household pets, commonly present lower infection rates, which may be due to a diet comprising mainly of cooked food or kitchen scraps [24] that are unlikely to contain viable hydatid cysts. However, such differences in relative infection rates may also be explained by the fact that dogs which are allowed to roam free are less likely to receive regular anthelmintic treatment than, for example, dogs kept as pets or guard dogs [32].

Multiple studies have found that *E. granulosus* prevalence and/or abundance is higher in young dogs compared to adults [21], [22], [24], [34], supporting the hypothesis that protective immune responses increase with the age of the host [121]. However, changes in infection pressure due to behavioural differences related to dog's age cannot be ruled out [122]. In addition, prevalence studies have observed higher numbers of infected male dogs compared to females [22], [27]. A plausible reason might be that male dogs tend to break away from the pack and explore larger areas than females, due to their tendency towards territorial behavior and to go hunting [12].

Human behavior has also been recognized as playing a key role in the perpetuation of echinococcosis transmission [123]. This behaviour is closely related to human cultural and economic backgrounds [124]. The use of epidemiological techniques and anthropologic knowledge has served in the past to highlight the reasons for the distribution of echinococcosis [125]. Studies in Table S4 that reported dog owners' ethnicity as being related with canine infection rates also found a higher number of dogs per owner, lower levels of education and lower standards of animal care, when compared with other ethnic groups [36], [37]. Thus, this variable may act as a confounder for other risk practices. Likewise, the changes in agricultural practices following the collapse of the Soviet Union may partly explain the increase in echinococcosis in Central Asia [3]. The social and economic changes brought after the collapse of socialist administration, such as the return to small private farms, the proliferation of the clandestine slaughter or the lack of anthelmintic dog treatment, are associated with a substantial increase in echinococcosis [25].

There are numerous studies reporting high parasite prevalences in wild canids [126], [127], although none of these reported statistically significant associations with potential disease determinants. For instance, *E. granulosus* was a frequent helminth parasite found in wolves (*Canis lupus*) presenting a meta-prevalence above 19%, although the tapeworm was more commonly reported in the Nearctic wolf populations compared to the Palaearctic [128]. The predator-prey relationship between wolves and moose (*Alces alces*) in North America has been documented for a long time [129]. More recently, Joly and Messier suggested that *E. granulosus* might have an influence in the regulation of the intermediate host populations by increasing the risk of predation of heavily infected moose by wolves [130]. In North America, *E. granulosus* has not only been reported in wolves but also in coyotes (*Canis latrans*) [127]. In Kazakhstan, a prevalence of 19.5% (95%CI 8.8–34.9) has recently been reported in wolves [131]. In Australia, *E. granulosus* is widespread in wild dogs (dingoes (*Canis lupus f. dingo*) and dingo/domestic dog hybrids) and is occasionally seen in foxes (*Vulpes vulpes*) [126]. In Africa infections have been found in golden jackals (*Canis aureus*), silver backed jackals (*Canis mesomelas*) and African wild dogs (*Lycaon pictus*) [126]. Additionally, there is experimental evidence of successful transmission between wild and domestic hosts [132]. Thus, wild hosts represent an important reservoir for *E. granulosus* transmission particularly where there is an overlap between human and wild animal habitats [133].

A wide range of domestic ungulates such as sheep, goats, cattle, pigs, equines and camelids serve as intermediate hosts for the larval stage (metacestode) of *E. granulosus*. The majority of risk factor studies in livestock species reported cross-sectional data from abattoir surveys. Environmental temperature and humidity are major influencing factors for livestock infection [134]. Low temperatures and high rainfall permit longer viability of eggs in the environment, a critical factor when ensuring the perpetuation of the parasite cycle. Hence, several studies have reported higher levels of CE in domestic livestock in areas presenting these environmental conditions when compared with warmer and drier sites [38], [47], [135]. The age-dependent increment in infection rates has been reported in many studies supporting the apparent lack of parasite-induced immunity in naturally infected intermediate hosts [134]. Therefore, both prevalence and abundance of hydatid cysts increase with age in intermediate hosts [134]. Alternatively, particular husbandry practices associated with age could explain the large prevalence reported in some farm species, like cattle and camels in Ethiopia [48].

Experimental studies have suggested that parasite survival may be longer in females due to the potential link between sexual hormones and the response of the immune system [136]. In Ethiopia male small ruminants were reported with higher infection risk compared to female [51], although this study may be biased as larger numbers of males than females were included in the sampled population. An alternative explanation may lie in the fact that females are slaughtered at older age as they are retained for reproductive purposes [47], [54]. Therefore, a longer life expectancy increases the probability of exposure and infection. Consequently, higher prevalences are usually found in older animals [54], [137].

Sheep frequently present the highest infection rate [54], [138] and are often the most important intermediate hosts for *E. granulosus* [2]. However, cattle and camels are normally sent to the abattoir at an older age than other ruminants, and hence have an increased risk of exposure to *E. granulosus'* eggs during their lifetime. Goats show lower infection rates, possibly because they are browsers and eat the most distal parts of plants where there are fewer eggs. Moreover, these eggs commonly have a greater exposure to hostile environmental conditions, and thus show a reduced infective capacity [139]. The difference in prevalence between host species could also be a result of the existence of different strains of *E. granulosus* morphologically and biochemically adapted to each farm species [48]. Human activities play also a critical role in the persistence of *E. granulosus* in farm species. Different management practices might be behind the infection differences showed between family and industrial pig farms [50], [68]. Similarly, the local cattle breeds in Ethiopia presented higher infection rates than the crossbreeds presumably because crossbreeds are frequently kept indoors whereas local breeds are pasture-grazing animals [67]. In Sardinia, the highest sheep prevalences were associated with farms whose owners admitted throwing the viscera into the trash/garbage and feeding their dogs with offal [140].

Wild animals can also act as intermediate host for *E. granulosus*. In North America, hydatid cysts have been found in elk (*Cervus canadensis*), moose (*Alces alces*), red deer (*Cervus elaphus*), caribou (*Rangifer tarandus*) and various species of deer [127]. In Canada, researchers have reported an age-related hydatid prevalence and intensity; suggesting the absence of immunity in wild intermediate hosts [70], [71]. In the same region, *E. granulosus* infection in moose was also related with increasing population density. Authors suggested that higher numbers of moose were linked with a more intense wolf predation pressure, and hence these moose were exposed to a higher environmental parasitic contamination [70]. In Africa, herbivores such as warthogs (*Phacochoerus sp.*), hippopotamus (*Hippopotamus amphibius*), giraffes (*Giraffa camelopardalis*), zebras (*Equus quagga*, *Equus zebra*) or impalas (*Aepyceros melampus*) are known to be susceptible to CE [141]. In Australia, CE has been reported in native mammals belonging to the Macropodidae family, such as kangaroos (*Macropus giganteus*, *Macropus fuliginosus*) and wallabies (*Wallabia bicolor*, *Macropus rufogriseus*), along with other marsupials such as wombats (*Vombatus ursinus)* [133]. The higher hydatid infection and intensity showed in eastern grey female kangaroos compared to males were suggested to be age-related and attributed to a higher human hunting pressure on larger animals, older males preferentially. Thus, female kangaroos live longer and hence are more likely to present higher infection and intensity rates than males [69].

*E. multilocularis* is endemic in foxes in large areas over the northern hemisphere [17]. In humans the larval stage of *E. multilocularis* causes AE, a space-occupying lesion, which is lethal if untreated. Association between parasite infection/burdens and young age in foxes have been frequently reported [74]–[76]. Nevertheless, differences in prevalence between juveniles and adults have not always been statistically significant [77], [101]. Investigators have not arrived to a conclusive biological reason for finding juveniles more frequently and/or intensively infected than adults. A proposed explanation is that adult foxes might acquire partial immunity after repeated exposure [75], [76] and young foxes could be more susceptible to infection when they assume a similar diet to that of the adults [81]. Endemic levels might also contribute to the differences in prevalence reported by host age [81] as low infection pressure can lead to an upward shift of the age at which protective immunity is acquired. This is known as “the peak shift” [142]. Only one study was found reporting a significant association between fox gender and parasite abundance. Nevertheless, male foxes tend to expand their territories further than females, and thus, they can play a significant role in dispersing the parasite when they are heavily infected [76].

The spatial distribution of *E. multilocularis* infection in foxes comes as a result of a combination of multiple ecological factors. Landscape features and regional climatic conditions not only affect the viability of *E. multilocularis* eggs in the environment but also shape the type of biodiversity given in a region, such as intermediate host populations, which determines parasite transmission. In France, the percentage of grassland was associated with fox coproantigen distribution, possibly related with sudden large increases in rodent populations known to occur in these areas [97]. Additionally, intensive land-use may lead to lower levels of water in the soil hampering the survival of parasitic eggs in the environment [81] whilst regions with high levels of soil humidity (e.g. pastures) present favourable conditions for the survival of the oncospheres outside the host [98].

Regional meteorological conditions contribute significantly to the spatial patterns of infection in foxes. *E. multilocularis* eggs are highly sensitive to both desiccation and high temperatures [143]. Consequently, infected foxes are more frequently found in areas with humid conditions [98]. Similarly, seasonal variations in temperature and precipitation influence the availability of definitive and intermediate hosts and the survival of the parasitic eggs in the environment. This seasonal prevalence fluctuation has been found related with factors such as the host's age [75], [82].

Transmission dynamics of *E. multilocularis* depend directly on the predator-prey relationship of their two hosts [10], which in turn respond to environmental conditions among other ecological factors. Local geographic and climatic conditions affect fox and rodent densities, resulting in marked spatial differences in parasite distribution among regions and seasons [75]. In Germany infected foxes were more frequently caught near humid areas and pastures that not only permit survival of oncospheres but also offer a suitable habitat for muskrats (*Ondatra zibethicus*), a susceptible intermediate host [98].

Furthermore, changes in fox population demographics can come as a result from human-related activities, like the progressive expansion of urban areas. In the UK, the increase of fox densities in some cities is believed to be a consequence of the construction of large residential suburbs highly suitable for foxes [144]. The same trend has also been reported in several European cities following the fox population growth after the successful vaccination campaign against rabies [76], [145]. Some other suggested factors responsible for this phenomenon are the greater availability of food (anthropogenic food), the availability of shelter and the lower hunting pressure found in human settlements [145], [146]. Moreover, high infection rates of *E. multilocularis* have been recorded in foxes close to urban settlements [76], [102]. The increase of fox densities together with the high parasite rates found in foxes near to the edges of cities might have resulted in higher environmental contamination [146]. However, this potential risk of infection may not be of importance as low prevalences in foxes have been reported in city centres compared to peri-urban or rural foxes [78], [101]. The scarcity of suitable intermediate prey-hosts in the urban centers and the increased availability of anthropogenic food might have contributed to this low infection rate [82], [101].

In addition to foxes, other members of the family Canidae, such as domestic dogs (*Canis lupus f. familiaris*), wolves (*Canis lupus*), coyotes (*Canis latrans*) or raccoon-dogs (*Nyctereutes procyonoides*), are also susceptible to be infected by *E. multilocularis* [147]. Likewise, some members of the family Felidae, such as wildcats (*Felis silvestris*) or domestic cats (*Felis silvestris f. catus*), can harbour *E. multilocularis* worms, although, cats appear to be a poor host for *E. multilocularis* [147]. In contrast, domestic dogs are an important definitive host and may contribute to the maintenance of *E. multilocularis* in a synanthropic cycle, particularly in certain rural communities [148]. The presence of *E. multilocularis* in dogs has been previously reported in endemic areas [12], [149]. Some of the risk factors associated with the acquisition of *E. multilocularis* are similar to those found for *E. granulosus*, such as non-restrained dogs or being a dog fed with uncooked viscera [12], [108]. As with *E. granulosus*, untied dogs have more possibilities of hunting small mammals and, thus have greater exposure to infection [12], [109]. Positive coproantigen results were mainly reported in working dogs such as hunting, guard or shepherd dogs [108] that presumably are more likely to roam freely and less likely to be dewormed regularly. The high numbers of positive dogs found in southern Germany might be related with high parasite prevalences presented in fox populations in the same region [110]. The role of domestic dogs in the transmission of *E. multilocularis* to humans appears to be of importance in certain communities where dog ownership, number of dogs owned or contact with them were found associated with human AE risk [14], [150].

The predator-prey dynamics between definitive and intermediate hosts are a key determinant driving *E. multiocularis* transmission [113]. This relationship depends on the host population densities and structures, which are directly influenced by ecological interacting factors such as availability of food, dispersion, reproduction and survival trends [151]. Rodent species are often found in specific landscapes, such as grassland areas, where food and cover are abundant. A hypothesis suggests that the ratio of these optimal habitats can influence the probability of arvicolids undergoing multi-annual cycles [152]. High prevalences of *E. multilocularis* have been reported in foxes in areas presenting a high ratio of grassland [113]. Hence, landscape characteristics contribute to population dynamics of arvicolid species and predator–prey interactions, and ultimately may influence parasite transmission [153]. The risk of *E. multilocularis* infection in rodents is also reliant on local meteorological conditions [143]. Additionally, vole populations commonly present a seasonal reproduction pattern starting in early spring and continuing until later into the autumn. Similarly, their age-structure is also closely dependent to seasonal oscillations, showing a higher proportion of adult voles in spring due to the decreased reproduction during winter [116]. Several studies reported an increasing prevalence of *E. multilocularis* in rodents with age. Therefore, seasonal variations of prevalence in rodents result from shifts in the age structure of voles' populations since a higher number of intermediate hosts are potentially harbouring protoscoleces during winter and beginning of spring [116]. The availability of prey affects the prevalence of *E. multilocularis* in definitive hosts [82], [107], [118]. Conversely, the number of foxes determines the level of environmental egg contamination in an area, and thus influences the infection rates in small mammals. For instance, in Geneva (Switzerland) low numbers of infected *A. terrestris* were captured in the south-eastern area of the canton where the fox population had decreased due to sarcoptic mange, suggesting that a lower environmental faecal contamination of parasitic eggs might explained the low infection rates recorded in rodents [117].

CE continues to represent a global health hazard affecting approximately over 1 million individuals worldwide [18]. Principal factors reported in this review to be associated with canine infection included potential access of dogs to uncooked livestock viscera, to be an unrestrained young and/or male dog and particular human activities linked with poor health education and living conditions of dog owners. Hence, some recommended measures to interrupt parasite transmission encompass controlled slaughtering of livestock and proper disposal of offal, regular treatment of dogs with praziquantel, vaccination of intermediate hosts and an improvement to the level of health education in poor rural livelihoods [154].

Although AE is confined to the northern hemisphere and generally is a less common disease than CE, is an often-fatal condition when untreated [155]. In addition, the increasing prevalence detected in wild life accompanied by the movement of foxes towards urban areas increases the risk for transmission to humans in Europe [146]. With a complex life cycle involving wildlife hosts, control of *E. multilocularis* remains challenging. Some of the reported ecological factors in this review affecting the transmission dynamics of *E. multilocularis* are hosts' population densities, predator-prey interactions, landscape characteristics, climate conditions and human-related activities. Current control strategies mainly focus on decreasing prevalence on definitive hosts through the distribution of anthelminthic baits for foxes or regular deworming of domestic dogs and preventing infection through education campaigns [154].

The burden of endemic neglected zoonoses falls heavily on rural settings with limited resources [156]. Livestock-rearing communities with subsistence-farming practices are high-risk areas for acquiring CE, while the vast majority of human AE cases are found in certain rural communities in China. Poor health services and shortage of equipment and drugs constrain the diagnosis and treatment of cases, causing premature death or health disabilities. Therefore, it is critical to prevent infection to reduce human incidence. Control of echinococcosis currently relies on the interruption of parasite transmission in animal hosts and, in consequence, a sound understanding of infection risk factors in animals can effectively assist the drawing of a prevention plan. Quantitative frameworks, such as the use of mathematical models, are of great value in the epidemiological research and control of *Echinococcus spp*. in a cost-effective way. This systematic review provides a compilation of epidemiologic factors associated with *Echinococcus* infection in animal hosts identified by the use of associative statistical models in order to assist the design of sound control policies.

## Supporting Information

Checklist S1 PRISMA checklist.
(PDF)

Table S1 Glossary of statistical terms.
(PDF)

Table S2 Studies assessing association between *E. granulosus* infection in dogs and potential access to raw offal.
(PDF)

Table S3 Studies identifying significant associations of age/gender and infection of dogs with *E. granulosus*.
(PDF)

Table S4 Studies assessing association between *E. granulosus* infection in dogs and socio-economic factors.
(PDF)

Table S5 Associative studies of *E. granulosus* infection in intermediate hosts.
(PDF)

Table S6 Associative studies of *E. granulosus* infection in wild intermediate hosts.
(PDF)

Table S7 Studies identifying significant determinants of infection of foxes with *E. multilocularis.*
(PDF)

Table S8 Studies assessing association between *E. multilocularis* infection in foxes and environmental factors.
(PDF)

Table S9 Spatial studies of *E. multilocularis* in foxes.
(PDF)

Table S10 Studies assessing association between *E. multilocularis* infection in foxes and host population factors.
(PDF)

Table S11 Associative studies of *E. multilocularis* infection in carnivores, other than foxes.
(PDF)

Table S12 Associative studies on *E. multilocularis* infection in intermediate hosts.
(PDF)
