# Supplementary material for: A Systematic Review of the Epidemiology of Echinococcosis in Domestic and Wild Animals
Source: PLoS Negl Trop Dis. 2013 Jun 6;7(6):e2249. doi: 10.1371/journal.pntd.0002249 (PMC3674998; doi:10.1371/journal.pntd.0002249)
Supplement: Table S1 — Glossary of statistical terms. (PDF) [file pntd.0002249.s002.pdf]

Table S1. Glossary of statistical terms

Term	Definition
Akaike information criterion (AIC)	A measure of the relative goodness of fit of a statistical model. The second-order Akaike Information Criterion (AICc) is equivalent to AIC but with a correction for finite sample sizes. The Akaike difference Delta (ΔAICc) shows the difference between the model AICc and the lowest AICc for the model set. The AICc weights help to valuate the relative importance of each variable. Lower AIC/AICc, lower ΔAICc or higher AICc weight indicate good plausible models and viceversa
Analysis of variance	Statistical technique which assesses the effect of categorical explanatory variables on the response variable
Binomial logistic regression	Statistical method to analyse the association between a binomial response variable (such as presence/absence of infection) and one or more explanatory variables. In such logistic regression the response variable is transformed to the logistic scale
Coefficient of determination (R^2)	Proportion of variation in the response variable explained by the statistical model
Conditional autoregressive spatial modelling	Type of regression model that captures spatial dependency and provides information on spatial relationships among the variables modeled
Conditional logistic regression	Type of logistic regression that analyses a categorical response variable when observations are not independent but matched or grouped in some way
Confidence intervals (CI)	Intervals that provide a range of values for a variable of interest constructed so that if the experiment can be repeated many times, the value of the parameter will lie within this interval in a confidence level of occasions, usually set to 95%
Correlation coefficient	Determines the degree of linear relationship between two variables
Cross-sectional	Epidemiologic study involving the observation of a population, or a

study	representative subset, at one specific point in time
Determinant	Any factor or variable that can affect the frequency with which a disease occurs in a population
Explanatory variable	A factor or exposure that may influence the occurrence of the response variable
Fixed-effects model	Type of model that treats the varying coefficients of explanatory variables as if the quantities were non-random (constant over all groups)
Interaction effect	Situation when the effect of the explanatory variable(s) on the response variable depends on the value of another explanatory variable(s)
Linear regression	Estimates the best-fitting straight line to describe the linear relationship between two variables
Mixed-effects model	Type of multilevel model that includes both fixed and random effects
Multilevel models	Statistic models of parameters that vary at more than one level
Multivariable analysis	Statistic analysis that includes more than one explanatory variable and where any potential issues, such confounding and interaction, are taken into account
Multivariate analysis	Statistical analysis where there is more than one response variable regardless of the number of explanatory variables included
Negative binomial regression	Type of statistical model that assumes that the dependent variable has an aggregated distribution described by the negative binomial distribution
Odds ratio (OR)	Measure of association between exposure and disease. The OR represents the odds that disease will occur given a particular exposure, compared to the odds of disease occurring in the absence of it
Ordinal logistic regression	Type of logistic regression that analyses an ordinal categorical response variable (with categories that have some intrinsic order) with one or more explanatory variables
p -value	Probability of obtaining a test statistic at least as extreme as the one that was actually observed, assuming that the null hypothesis is true
Random-effects model	Type of multilevel model which varying coefficients of explanatory variables have a different value for each of the groups (or levels)

Response variable	Variable that is the focus of the analysis, whose variation is trying to be explained by the explanatory variables
Risk ratio (RR)	Measure of association between exposure and disease. The RR gives a ratio of disease risk in the exposed compared to disease risk in the non-exposed
Semivariogram	Function describing the degree of spatial dependence of a spatial random field or stochastic process
Spatial scan statistic	Statistical method to test for spatial clustering
Univariable analysis	Statistic analysis that includes just one explanatory variable
Univariate analysis	Statistic analysis that deals with a single response variable regardless of the number of explanatory variables included
Variable	Characteristic that can be measured in different individuals or groups capable of adopting different values
Zero inflated model	Type of count regression model when there are additional zero counts then can be described by a standard count model such as Poisson or negative binomial
