# Supplementary material for: A Systematic Review of the Epidemiology of Echinococcosis in Domestic and Wild Animals
Source: PLoS Negl Trop Dis. 2013 Jun 6;7(6):e2249. doi: 10.1371/journal.pntd.0002249 (PMC3674998; doi:10.1371/journal.pntd.0002249)
Supplement: Table S2 — Studies assessing association between E. granulosus infection in dogs and potential access to raw offal. (PDF) [file pntd.0002249.s003.pdf]

Table S3. Studies assessing association between *E. granulosus* infection in dogs and potential access to raw offal

Reference	Study Information	Statistical Method	Significant Factor
Bchir et al., 1987 [29]	Post-mortem inspection of 50 dogs in Central Tunisia	Univariable analysis	Dogs shot within 1 km of a refuse dump (p<0.001)
Parada et al., 1995 [27]	Arecoline purgation of 704 dogs in Durazno (Uruguay)	Univariable analysis	Dogs with access to fields (p<0.05) and not tied-up dogs (p<0.001)
Moro et al., 1999 [20]	Arecoline purgation of 63 dogs in central Peruvian Andes	Univariable analysis	Dog fed with hydatid-infected viscera were (p<0.004) and sheep-dogs were more likely to be infected (p<0.001)
Wang et al., 2001 [23]	Coproantigen examination of 139 owned dogs in Narenhebuke commune (China)	Univariable analysis	Dogs from the winter area presented higher coproantigen positivity than the ones from the summer pasture (p<0.01)
Shaikenov et al., 2003 [25]	Arecoline purgation of 2,071 dogs in Southern Oblasts (Kazakhstan)	Univariable analysis	Farm dogs present higher abundance of infection and prevalence (p<0.001)
Buishi et al., 2005 [24]	Coproantigen examination of 334 dogs in Tripoli (Libya)	Multivariable logistic regression	Sheep-dogs showed an increased risk of coproantigen positivity compared to household dogs (OR 9.791, 95%CI 1.081-88.66, p=0.042)
Buishi et al., 2005 [33]	Coproantigen examination of 1,164 farm dogs in Wales (UK)	Multivariable logistic regression	Unrestrained dogs had higher risk of coproantigen positivity (OR 2.91, 95%CI 1.77-4.8, p<0.0001)
Perez et al., 2006 [26]	Coproantigen examination of 748 dog faecal samples from livestock farms in Rio Negro (Argentina)	Univariable analysis	Dog prevalence increased with increasing number of dogs (p=0.0028) (i.e. OR 4.19, comparing having 1 dog with having ≥5) and sheep

			(p=0.0039) (i.e. OR 4.29, compared 0 with ≥ 2,501)
Buishi et al., 2006 [21]	Coproantigen examination of 161 dogs in Turkana (Kenya)	Multivariable logistic regression	Dogs fed with raw offal (OR 22.74, 95%CI 2.60-199.08, p=0.005) and dogs free to roam were at higher risk of being coproantigen positive (OR 14.56, 95%CI 2.70-78.50, p=0.002) whereas the proper disposal of carcases by dog-owners reduced such risk (OR 0.07, 95%CI 0.01-0.33, p=0.001)
El Shazly et al., 2007 [28]	Post mortem examination of 540 dogs in Dakahlia (Egypt)	Univariable analysis	Rural dogs compared to urban dogs (p=0.03)
Guzel et al., 2008 [30]	Coproantigen examination of 79 owned dogs in Antakya (Turkey)	Univariable analysis	Unrestrained dogs had increased risk of coproantigen positivity (p<0.05)
Huang et al., 2008 [31]	Coproantigen examination of 23 stray dogs and 580 owned dogs in Tibet (China)	Univariable analysis	Unrestrained dogs (p<0.01) compared to those tied during the day or/and night
Inangolet et al., 2010 [34]	Post mortem examination of 327 dogs in the Moroto District (Uganda)	Ordinal logistic regression	Stray dogs presented higher parasite burdens compared to domesticated dogs (OR 5.42, 95%CI 2.27–12.92), p<0.001)
Acosta-Jamett et al., 2010 [22]	Coproantigen examination of 334 dogs in Coquimbo (Chile)	Multivariable mixed-effects logistic regression	Dogs from households not practising home-slaughter (OR 0.04, 90%CI 0.01–0.13, p=0.001), from rural sites (OR 0.01, 90%CI 0.002–0.05, p=0.001) and with longer

			distance to rural areas (OR 0.01, 90%CI 0.001–0.17, *p*=0.007) showed lower prevalence
Mastin et al., 2011 [32]	Coproantigen examination of 577 dogs in South Powys (Wales)	Multivariable mixed-effects logistic regression	Dogs regularly roaming had higher coproantigen positivity (OR 4.93, 95%CI 1.87–13.00, *p*=0.001)

Measures of association reported when available
Abbreviations: OR, odds ratio; CI, confidence interval.
