# Supplementary material for: A Systematic Review of the Epidemiology of Echinococcosis in Domestic and Wild Animals
Source: PLoS Negl Trop Dis. 2013 Jun 6;7(6):e2249. doi: 10.1371/journal.pntd.0002249 (PMC3674998; doi:10.1371/journal.pntd.0002249)
Supplement: Table S3 — Studies identifying significant associations of age/gender and infection of dogs with E. granulosus. (PDF) [file pntd.0002249.s004.pdf]

Table S3. Studies identifying significant associations of age/gender and infection of dogs with *E. granulosus*

Reference	Study Information	Statistical Method	Significant Factor
Parada et al., 1995 [27]	Arecoline purgation of 704 dogs in Durazno (Uruguay)	Univariable analysis	Male dogs were more likely to be infected than females ($p<0.05$)
Sharifi et al., 1996 [35]	Post mortem examination of 6,500 dogs in Kerman (Iran)	Univariable analysis	Dogs from 0-2 years showed higher prevalence compared to dogs of 3-4 years ($p<0.05$) and >5 years ($p<0.002$)
Buishi et al., 2005 [24]	Coproantigen examination of 334 dogs in Tripoli (Libya)	Multivariable logistic regression	Dogs >5 years presented lower coproantigen positivity (OR 0.852, 95%CI 0.731-0.993, $p<0.04$)
Buishi et al., 2006 [21]	Coproantigen examination of 161 dogs in Turkana (Kenya)	Multivariable logistic regression	Dogs ≤5 years presented higher coproantigen-positive results (OR 0.47, 95% CI 0.29-0.76, $p=0.002$)
Acosta-Jamett et al., 2010 [22]	Coproantigen examination of 334 dogs in Coquimbo (Chile)	Multivariable mixed-effects logistic regression	Dogs > 2 years presented lower odds of being coproantigen-positive (OR 0.11, 90% CI 0.04–0.29, $p=0.001$)
Inangolet et al., 2010 [34]	Post mortem examination of 327 dogs in the Moroto District (Uganda)	Ordinal logistic regression	Dogs >5 years presented lower worm counts (OR 0.07, 95%CI 0.04–0.16, $p<0.001$)

Measures of association reported when available
Abbreviations: OR, odds ratio; CI, confidence interval.
