# Supplementary material for: A Systematic Review of the Epidemiology of Echinococcosis in Domestic and Wild Animals
Source: PLoS Negl Trop Dis. 2013 Jun 6;7(6):e2249. doi: 10.1371/journal.pntd.0002249 (PMC3674998; doi:10.1371/journal.pntd.0002249)
Supplement: Table S4 — Studies assessing association between E. granulosus infection in dogs and socio-economic factors. (PDF) [file pntd.0002249.s005.pdf]

Table S4. Studies assessing association between *E. granulosus* infection in dogs and socio-economic factors

Reference	Study Information	Statistical Method	Significant Factor
Burridge et al., 1977 [37]	Arecoline purgation of dogs to assess progress of hydatid control in New Zealand	Multivariable regression	Percentage of Maori people was negatively related with rate of progress in disease control (R^2=0.215) [1]
Pappaioanou et al., 1984 [36]	Arecoline purgation of dogs to assess progress of anti-echinococcosis campaign in Cyprus	Multivariable logistic regression	Ethnic village type was associated with higher dog prevalence (p<0.0001)
Parada et al., 1995 [27]	Arecoline purgation of 704 dogs in Durazno (Uruguay)	Univariable analysis	Dogs reported to be dewormed with praziquantel were less likely to be infected (p<0.01)
Buishi et al., 2005 [33]	Coproantigen examination of 1,164 farm dogs in Wales (UK)	Multivariable logistic regression	Dogs not dewormed frequently (>6 months) presented higher risk of coproantigen positivity (OR 3.16, 95%CI 1.46–6.85, p=0.004)
Buishi et al., 2005 [24]	Coproantigen examination of 334 dogs in Tripoli (Libya)	Multivariable logistic regression	Dog owners reporting lack of knowledge about hydatid disease presented an increased risk of having a positive coproantigen dog (OR 3.278, 95%CI 1.045-10.28, p=0.042)
Huang et al., 2008 [32]	Coproantigen examination of 23 stray dogs and 580 owned dogs in Tibet (China)	Univariable analysis	Dogs whose owners lacked hydatid transmission knowledge (p<0.05) and did not have de-worming practice (p<0.01)
Acosta-Jamett et al., 2010 [22]	Coproantigen examination of 334 dogs in Coquimbo (Chile)	Multivariable mixed-effects logistic regression	Households reporting not have been dewormed their dogs in the last 2 months presented higher prevalence (OR 5.23, 90%CI 1.98–13.8,

			p=0.005)

Measures of association reported when available

[1] The percentage of Maori people in the total population explained 21.5% of the variation in *E. granulosus* infection in dogs. No significant test is provided for the R^2 change.

Abbreviations: R^2, Coefficient of determination; OR, odds ratio; CI, confidence interval.
