# Supplementary material for: A Systematic Review of the Epidemiology of Echinococcosis in Domestic and Wild Animals
Source: PLoS Negl Trop Dis. 2013 Jun 6;7(6):e2249. doi: 10.1371/journal.pntd.0002249 (PMC3674998; doi:10.1371/journal.pntd.0002249)
Supplement: Table S5 — Associative studies of E. granulosus infection in intermediate hosts. (PDF) [file pntd.0002249.s006.pdf]

Table S5. Associative studies of *E. granulosus* infection in intermediate hosts

Reference	Study Information	Statistical Method	Significant Factor
Pandey et al., 1986 [53]	Abattoir survey of 2,246 sheep, 510 goats and 35 dromedaries in Morocco	Univariable analysis	Age increasing prevalence in sheep (p<0.01)
Abdul-Salam et al., 1988 [60]	Parasitological examination of 293 camels in Kuwait	Univariable analysis	Females presented higher cyst infection (p=0.015) compared to males
Ming et al., 1992 [62]	Parasitological examination of 2,106 sheep in the Xinjiang Uygur Autonomous Region (China)	Univariable analysis	Females presented higher cyst prevalence (p<0.0001)
Cabrera et al., 1995 [58]	Post-mortem examination of 501 sheep in Florida (Uruguay)	Univariable analysis	Difference between age groups in sheep (p<0.01)
Njoroge et al., 2002 [38]	Abattoir survey of 381 cattle, 588 sheep, 5,752 goats and 70 camels in Turkana (Kenya)	Univariable analysis	Geographic location (p<0.05)
Tashani et al., 2002 [54]	Abattoir survey of 614 cattle, 1087 sheep, 881 goats and 428 camels in Benghazi (Libya)	Univariable analysis	Age and prevalence correlated in sheep (p=0.042), cattle (p<0.001) and camels (p=0.053). Sheep had the highest hydatid infection (p<0.01) and goats the lowest (p<0.01). Higher prevalence in females than males (p<0.01)
Umur et al., 2003 [55]	Abattoir survey of 1,355 cattle, 218 sheep and 104 goats in Burdur (Turkey)	Univariable analysis	The prevalence and number of cysts increased with age (p<0.05)
Islam et al., 2003 [49]	Abattoir survey of 405 cattle, 142 sheep, 292 goats and 108 buffaloes in Cox's Bazar (Bangladesh)	Univariable analysis	Host species and age (p<0.001)
Sharma et al., 2004 [68]	Parasitological examination of 236 pig meat samples in	Univariable analysis	Higher prevalence in pigs reared in extensive conditions

	Punjab (India)		compared to intensive production ($p<0.05$)
Ahmadi et al., 2005 [39]	Abattoir survey of 661 camels in Iran	Univariable analysis	Geographic location ($p<0.005$)
Ansari-Lari, 2005 [46]	Retrospective abattoir study of 131,716 cattle, 577,090 sheep and 135,233 goats in Shiraz (Iran)	Univariable analysis	Seasonal variations in prevalence ($p<0.0001$)
Scala et al., 2006 [52]	Abattoir survey of 771 sheep in Sardinia (Italy)	Multivariable logistic regression	Cyst infection increased with host age (OR 1.15, 95%CI 1.0736– 1.2478, $p<0.0001$)
Azlaf et al., 2006 [40]	Abattoir survey of 618 cattle, 2,948 sheep, 2,337 goats, 482 camels and 455 equines in Morocco	Univariable analysis	Geographic origin and host species ($p<0.0001$)
Banks et al., 2006 [41]	Abattoir survey and retrospective abattoir data of 32,567 cattle in Queensland (Australia)	Multivariable logistic regression	Geographic origin and age ($p<0.01$)
Daryani et al., 2007 [61]	Abattoir survey of 928 cattle, 3,765 sheep, 445 goats and 243 buffaloes in Ardabil (Iran)	Univariate analysis	Female gender (sheep and cattle) ($p<0.001$) and seasonal prevalence patterns (sheep) ($p<0.001$)
Cringoli et al., 2007 [64]	Abattoir survey of 2587 cattle and 612 water buffaloes in the Campania (Italy)	Univariable analysis	Host species and sheep farms closer to cattle positive farms than water buffalo positive farms ($p<0.001$)
Lahmar et al., 2007 [43]	Ultrasound screening of 1,039 sheep in the northeast of Tunisia	Univariable analysis	Geographic origin ($p<0.01$) and age ($p<0.05$)
Christodoulopoulos et al., 2008 [56]	Abattoir survey of 700 hoggets and 1500 sheep in Thessaly (Greece)	Univariable analysis	Age ($p<0.001$)
Ernest et al., 2009 [45]	Retrospective abattoir study of 2,677 cattle and 607 sheep and 3,047 goats in Arusha (Tanzania)	Univariable analysis	Host species ($p<0.001$) and geographic location ($p<0.001$)
Bruzinskaite et al., 2009 [50]	Abattoir of 648 pigs in Southwestern (Lithuania)	Univariable analysis	Age ($p<0.01$) and type of farm ($p<0.02$)
Nonga et al., 2009 [63]	Retrospective abattoir	Univariable analysis	Sheep and goats

	study of 115,186 cattle and 99,401 sheep and goats in Arusha (Tanzania)		showed higher hydatid infection in 2005 (OR 2.2, $p<0.001$) and 2007 (OR 1.6, $p<0.001$) compared to cattle
Regassa et al., 2009 [42]	Abattoir survey of 415 cattle in Southern Ethiopia	Multivariable logistic regression	Host origin (OR 2.8, 95%CI 1.18, 6.51, $p=0.021$)
Kebede et al., 2009 [65]	Abattoir study of 420 cattle and 340 sheep in Bahir Dar (Ethiopia)	Univariable analysis	Host species ($p<0.001$)
Ibrahim, 2010 [47]	Abattoir survey of 2,668 cattle, 6,525 sheep, 3,578 goats and 140 camels in Al Baha (Saudi Arabia)	Multivariable logistic regression	Host species, age, gender (cattle and sheep) and seasonal variations (sheep and goats) ($p<0.05$)
Erbeto et al., 2010 [51]	Abattoir survey of 1,053 sheep and 639 goats in Addis Ababa (Ethiopia)	Univariable analysis	Host species, age, gender and type of production system ($p<0.05$)
Getaw et al., 2010 [66]	Abattoir survey of 852 cattle, 92 sheep and 208 goats in central Ethiopia	Univariable analysis	Host species ($p<0.001$)
Acosta-Jamett et al., 2010 [44]	Retrospective abattoir study of 174,034 cattle, 35,404 sheep, 22,208 goats, 25,355 pigs and 9,391 equines in Coquimbo (Chile)	Linear correlation (Spearman's rank coefficient)	Host species ($p<0.001$), geographic origin ($p<0.001$) and negative correlation between prevalence in goats and rainfall ($p=0.02$)
Zewdu et al., 2010 [59]	Abattoir survey of 384 zebu cattle in Ambo (Ethiopia)	Univariable analysis	Age ($p<0.0001$)
Bekele et al., 2011 [67]	Abattoir survey of 546 cattle in southern Ethiopia	Univariable analysis	Local breeds harboured higher infection levels ($p=0.043$)
Fromsa et al., 2011 [48]	Retrospective abattoir study of 22,863 cattle, 6,518 sheep, 1,753 goats, 417 camels and 150 pigs in Ethiopia	Univariable analysis	Host species ($p<0.001$), higher altitude (cattle) ($p<0.001$) and (sheep) ($p<0.01$)
Marshet et al., 2011 [57]	Abattoir survey of 611 sheep and 389 goats in Addis Ababa (Ethiopia)	Multivariable logistic regression	Host species (OR 5.14, 95%CI 2.76-9.55, $p<0.0001$) and age (OR 1.68, 95%CI 1.22-2.85, $p<0.029$)

Measures of association reported when available, except for Ibrahim (2010) due to the large number of odds ratios and corresponding confidence intervals calculated. Abbreviations: OR, odds ratio; CI, confidence interval.
