# Supplementary material for: A Systematic Review of the Epidemiology of Echinococcosis in Domestic and Wild Animals
Source: PLoS Negl Trop Dis. 2013 Jun 6;7(6):e2249. doi: 10.1371/journal.pntd.0002249 (PMC3674998; doi:10.1371/journal.pntd.0002249)
Supplement: Table S6 — Associative studies of E. granulosus infection in wild intermediate hosts. (PDF) [file pntd.0002249.s007.pdf]

Table S6. Associative studies of *E. granulosus* infection in wild intermediate hosts

Reference	Study Information	Statistical Method	Significant Factor
McNeill et al., 1987 [71]	Parasitological examination of 580 lungs of moose (*Alces alces*) in southwestern Quebec (Canada)	Univariable analysis	Cyst intensity increased with moose age (p<0.01)
Messier et al., 1989 [70]	Parasitological examination of 232 lungs of moose (*Alces alces*) in southwestern Quebec (Canada)	Univariable analysis	Cyst prevalence and infection increased with moose density (p<0.01) and were correlated with moose age (p<0.01)
Barnes et al., 2007 [69]	Post mortem examination of 2,998 macropodids in Queensland (Australia)	Multivariable logistic regression	Eastern grey kangaroos females harbour more cysts than males (p=0.008)

Measures of association reported when available.
