# Supplementary material for: A Systematic Review of the Epidemiology of Echinococcosis in Domestic and Wild Animals
Source: PLoS Negl Trop Dis. 2013 Jun 6;7(6):e2249. doi: 10.1371/journal.pntd.0002249 (PMC3674998; doi:10.1371/journal.pntd.0002249)
Supplement: Table S7 — Studies identifying significant determinants of infection of foxes with E. multilocularis. (PDF) [file pntd.0002249.s008.pdf]

Table S7. Studies identifying significant determinants of infection of foxes with *E. multilocularis*

Reference	Study Information	Statistical Method	Significant Factor
Tackmann et al., 1998 [81]	Post mortem examination of 4,374 foxes in Brandenburg (Germany)	Univariable analysis	Under high endemic conditions juveniles were found more frequently infected than adults (p<0.001)
Morishima et al., 1999 [80]	Coproantigen detection and egg examination of 534 feacal samples of foxes in Hokkaido (Japan)	Univariable analysis	Juveniles (p<0.001) presented higher coproantigen positivity
Hofer et al., 2000 [76]	Post mortem examination of 388 red foxes in in Zurich (Switzerland)	Univariable analysis	Sub-adults (p<0.05) presented higher parasite burdens
Yimam et al., 2002 [77]	Post mortem examination of 67 red foxes in Otaru (Japan)	Univariable analysis	Juveniles (p<0.021) presented higher parasite burdens
Losson et al., 2003 [74]	Post mortem examination of 709 foxes in Wallonia (Belgium)	Univariable analysis	Juveniles (p=0.01) presented higher prevalence
Fischer et al., 2005 [78]	Post mortem examination of 267 foxes in Geneva (Switzerland)	Multivariable logistic regression	Juveniles (p=0.013) presented higher parasite burdens
Hegglin et al., 2007 [82]	Post mortem examination of 582 foxes in Zurich (Switzerland)	Multivariable logistic regression	Season * age (marked in juveniles) (AICc weight=0.69) and zone * age (marked for adults) (AICc weight=0.45) [1]
Brossard et al., 2007 [75]	Post mortem examination of 3,793 foxes in western Switzerland	Univariable analysis	Juveniles (p<0.001) presented higher prevalence and infection intensity
Ziadinov et al., 2010 [79]	Post mortem examination of 151 foxes in central Kyrgyzstan	Zero-inflated negative binomial model	Prevalence decreased with age (OR 1.28, 95%CI 1.01-1.62, p=0.042)
Bruzinskaitt-Schmidhalter et al., 2012 [83]	Post mortem examination of 310 red foxes in Lithuania	Multivariable logistic regression	Regressor parameters “juvenile” (95%CI [-0.54, -0.94]) and “male” (95%CI [1.20, 1.27]) were associated with parasite

			abundance

Measures of association reported when available

(*) Interaction term.

[1] The model explaining best the prevalence rate in foxes (lowest AICc) included the variables *Zone, season, age, zone × age, season × age*.

Abbreviations: OR, odds ratio; CI, confidence interval; AICc, Akaike's information criterion corrected for small samples sizes.
