# Supplementary material for: A Systematic Review of the Epidemiology of Echinococcosis in Domestic and Wild Animals
Source: PLoS Negl Trop Dis. 2013 Jun 6;7(6):e2249. doi: 10.1371/journal.pntd.0002249 (PMC3674998; doi:10.1371/journal.pntd.0002249)
Supplement: Table S8 — Studies assessing association between E. multilocularis infection in foxes and environmental factors. (PDF) [file pntd.0002249.s009.pdf]

Table S8. Studies assessing association between *E. multilocularis* infection in foxes and environmental factors

Reference	Study Information	Statistical Method	Significant Factor
Kritsky et al., 1978 [92]	Post mortem examination of 1,153 foxes in North Dakota (EE.UU)	Univariable analysis	Seasonal variation of prevalence (p=0.0131)
Tackmann et al., 1998 [81]	Post mortem examination of 4,374 foxes in Brandenburg (Germany)	Univariable analysis	Variations in prevalence among 3 geographic zones (p<0.001)
Hofer et al., 2000 [76]	Post mortem examination of 388 red foxes in in Zurich (Switzerland)	Univariable analysis	Seasonal variation in prevalence in urban sub-adult males (p<0.001)
Raoul et al. 2001 [88]	Post mortem examination of 222 red foxes in in Franche-Comté (France)	Univariable analysis	Higher prevalence found in mid-altitude areas compared to low altitude areas (p<0.001)
Denzin et al., 2005 [91]	Post mortem examination of 1,341 red foxes in Saxony-Anhalt	Multivariable logistic regression	Negative association with probability of infestation and the average annual maximum temperature (p=0.00001)
König et al. 2005 [84]	Post mortem examination of 268 foxes in Bavaria (Germany)	Univariable analysis	Variations in prevalence among 3 geographic areas (p<0.001)
Miterpáková et al., 2006 [89]	Parasitological examination of 3,096 foxes in Slovakia	Simple correlation	Prevalence (p=0.021) and abundance (p=0.020) correlated with mean annual precipitation
Dubinsky et al., 2006 [85]	Parasitological examination of 392 foxes in Poland	Univariable analysis	Higher prevalence in the Polish border area with Slovakia (p=0.0009)
Hegglin et al., 2007 [82]	Post mortem examination of 582 foxes in Zurich (Switzerland)	Multivariable logistic regression	Season (AICc weight=1) (i.e. summer/autumn vs winter, OR 0.78, 95%CI 0.38-1.61) and season * age (marked in juveniles) (AICc weight=0.69) [1]
Brossard et al., 2007	Post mortem	Univariable analysis	Variations in

[75]	examination of 3,793 foxes in western Switzerland		prevalence among geographic areas and seasons depending on host age ($p<0.05$)
Hanosset et al., 2008 [93]	Post-mortem examination of 990 foxes in Wallonia (Belgium)	Univariable analysis	Seasonal variations in prevalence. Summer/autumn, vs. winter/spring (OR 1.4, 95%CI 1.04–1.98, $p=0.03$)
Immelt et al., 2009 [87]	Post mortem examination of 959 foxes in South Hesse and Middle Hesse (Germany)	Multivariable logistic regression	Higher parasite burdens associated with areas with high agriculture land and high amount of precipitation ($p<0.0001$)
Miterpáková et al., 2009 [90]	Post mortem examination of 4,026 foxes in the Slovak Republic	Simple correlation and multivariable logistic regression	Correlation between the mean annual precipitation and both prevalence ($p=0.022$) and worm burden ($p=0.021$). Regional differences in prevalence ($p<0.001$)
Casulli et al., 2010 [86]	Post-mortem examination of 840 foxes in Hungary	Univariable analysis	Prevalence and abundance higher in the north-western half than in the south-eastern half of the country ($p<0.001$)

Measures of association reported when available
(*) Interaction term.
[1] The model explaining best the prevalence rate in foxes (lowest AICc) included the variables *Zone, season, age, zone × age, season × age*.
Abbreviations: OR, odds ratio; CI, confidence interval; AICc, Akaike's information criterion corrected for small samples sizes.
