# Supplementary material for: A Systematic Review of the Epidemiology of Echinococcosis in Domestic and Wild Animals
Source: PLoS Negl Trop Dis. 2013 Jun 6;7(6):e2249. doi: 10.1371/journal.pntd.0002249 (PMC3674998; doi:10.1371/journal.pntd.0002249)
Supplement: Table S9 — Spatial studies of E. multilocularis in foxes. (PDF) [file pntd.0002249.s010.pdf]

Table S9. Spatial studies of *E. multilocularis* in foxes

Reference	Study Information	Statistical Method	Significant Factor
Berke, 2001 [94]	Choropleth mapping of regional prevalence estimates based on parasitological examination from 5,365 foxes in Lower Saxony (Germany)	Conditional autoregressive spatial modelling	Raised prevalence in the southern and northern parts modeled with a second-order polynomial model ($p \leq 0.05$)
Staubach et al., 2001 [98]	Spatial analysis of infection status of 3,521 foxes on the background of geographic vector data in Brandenburg (Germany)	Univariable analysis	Infected foxes were collected closer to water bodies ($p=0.0048$), areas of high soil humidity ($p=0.013$) and on pastures ($p=0.078$)
Berke et al., 2002 [95]	Spatial analysis of parasitological examination of 5,365 red foxes in Lower Saxony (Germany)	Spatial scan statistic	Identification of disease cluster area from 1991-1997 (RR 4.80, 95%CI 4.11-5.63, $p=0.001$)
Pleydell et al., 2004 [97]	Spatial investigation of coproantigens patterns of 345 faecal samples from foxes in the Franche-Comté region (France)	Non-linear regression and semivariogram	The inclusion of the grassland index improved consistently the fitting of the models ($p<0.05$)
Denzin et al., 2005 [91]	Post mortem examination of 1,341 red foxes in Saxony-Anhalt	Spatial scan statistic	Identification of a clusters with increased risk of infection (RR 4.4, 95%CI 2.6-5.0, p=0,001)
Berke et al., 2008 [96]	Spatial-temporal analyses of parasitological examination of 8,459 foxes in Lower Saxony (Germany)	Spatial scan statistic	Fox infection was clustered in the southern part ($p \leq 0.01$)
Fuglei et al., 2008 [99]	Spatial coproantigen investigation of 473 artic fox faecal samples from Svalbard (Norway)	Estimation of fox feces densities by line transect methods and score confidence limits for the proportions	Highest proportion of fox coproantigen positive feces overlapped voles' geographical range ($p \leq 0.05$)

Measures of association reported when available
Abbreviations: RR, risk ratio; OR, odds ratio; CI, confidence interval; AIC, Akaike information criterion.
