# Supplementary material for: A Systematic Review of the Epidemiology of Echinococcosis in Domestic and Wild Animals
Source: PLoS Negl Trop Dis. 2013 Jun 6;7(6):e2249. doi: 10.1371/journal.pntd.0002249 (PMC3674998; doi:10.1371/journal.pntd.0002249)
Supplement: Table S10 — Studies assessing association between E. multilocularis infection in foxes and host population factors. (PDF) [file pntd.0002249.s011.pdf]

Table S10. Studies assessing association between *E. multilocularis* infection in foxes and host population factors

Reference	Study Information	Statistical Method	Significant Factor
Saitoh et al., 1998 [103]	Post mortem examination of 9,828 red foxes in Hokkaido (Japan)	Multivariable logistic regression	Vole abundance affected infection rates in foxes (p<0.001)
Hofer et al., 2000 [76]	Post mortem examination of 388 red foxes in in Zurich (Switzerland)	Univariable analysis	Higher prevalence in foxes from rural areas (p<0.01) vs. urban areas during winter
Stieger et al., 2002 [102]	Coproantigen examination of 604 fox faecal samples in Zurich (Switzerland)	Univariable analysis	Higher positive results in border and peri-urban zone compare to urban zone during winter (p<0.01)
Raoul et al., 2003 [105]	Coproantigen examination of 156 fox faecal samples in Le Souillot (France)	Univariable analysis	Decrease of infection as fox numbers reduce (p=0.0004)
Fischer et al., 2005 [78]	Post mortem examination of 267 foxes in Geneva (Switzerland)	Multivariable logistic regression	Level of urbanization. Rural vs urban (OR 2.73, 95%CI 1.24-5.97, p=0.012) and border vs urban (OR 2.32, 95%CI 1.03-5.18, p=0.04)
Tanner et al., 2006 [104]	Post mortem examination of 543 foxes in Grisons (Switzerland)	Linear correlation (Spearman's rank coefficient)	Prevalence correlated with predation on intermediate hosts (*Microtus/Pitymys*) (p=0.018)
Miterpáková et al., 2006 [89]	Parasitological examination of 3,096 foxes in Slovakia	Simple correlation	Prevalence correlated with density of small mammals (p=0.022)
Reperant et al., 2007 [100]	Post mortem examination of 228 red foxes in Geneva (Switzerland)	Multivariable logistic regression	Decrease prevalence from rural to urban areas (p=0.037)
Hegglin et al., 2007 [82]	Post mortem examination of 582 foxes in Zurich (Switzerland)	Multivariable logistic regression	Type of urbanization zone (AICc weight=1) (i.e. Border vs. peri-urban, OR 0.46 95%CI 0.25-0.85) and zone * age (marked for adults) (AICc weight=0.45) [1]
Hanosset et al., 2008	Post mortem	Linear correlation	Positive correlation

[93]	examination of 990 foxes in Wallonia (Belgium)	(Spearman's rank coefficient)	between prevalence in foxes and muskrats (Spearman's rank correlation coefficient=1, $p<0.0001$)
Robardet et al., 2008 [101]	Post mortem examination of 127 red foxes in Nancy (France)	Multivariable logistic regression	Type of urbanization zone (AICc weight=0.94). Urban vs. rural (OR 0.04, 95%CI 0.01-0.14) and peri- urban vs. rural (OR 0.38, 95%CI 0.14-1.01).

Measures of association reported when available
(*) Interaction term.
[1] The model explaining best the prevalence rate in foxes (lowest AICc) included the variables *Zone, season, age, zone × age, season × age*.
Abbreviations: OR, odds ratio; CI, confidence interval; AICc, Akaike's information criterion corrected for small samples sizes.
