# Supplementary material for: A Systematic Review of the Epidemiology of Echinococcosis in Domestic and Wild Animals
Source: PLoS Negl Trop Dis. 2013 Jun 6;7(6):e2249. doi: 10.1371/journal.pntd.0002249 (PMC3674998; doi:10.1371/journal.pntd.0002249)
Supplement: Table S11 — Associative studies of E. multilocularis infection in carnivores, other than foxes. (PDF) [file pntd.0002249.s012.pdf]

Table S11. Associative studies of *E. multilocularis* infection in carnivores, other than foxes

Reference	Study Information	Statistical Method	Significant Factor
Budke et al., 2005 [12]	Arecoline purgation of 371 owned dogs in the Tibetan Plateau (China)	Multivariable logistic regression	Dogs being allowed to roam were more likely of be infected (OR 0.3693, 95%CI 0.1593–0.8558, *p*=0.02)
Wang et al., 2007 [106]	Collection of faecal samples and arecoline purgation of 252 dogs in the Shiqu County (China)	Multivariable conditional logistic regression	Canine infection related with the density of small mammal burrows in the open pastures (OR=1.048, *p*=0.003)
Ziadinov et al., 2008 [109]	Arecoline purgation of 466 owned dogs in At-Bashy (Kyrgyzstan)	Multivariable logistic regression	Dogs being allowed to roam (OR 0.39, 95%CI 0.199– 0.749, *p*=0.0056) and hunting dogs (OR 4.2, 95%CI 1.89–9.68, *p*=0.0005) were more likely of be infected
Dyachenko et al., 2008 [110]	Cross-sectional survey of faecal samples of 17,894 dogs and 9,064 cats in Germany	Univariable analysis	Higher dog prevalence found in the south compared with the north (OR 2.6, 95%CI 1.4-4.9, *p*<0.01)
Antolova et al., 2009 [108]	Coprological examination of 289 dogs in Slovakia	Multivariable logistic regression	Dogs being fed with raw offal (OR 7.05, 95%CI 1.24-40.09, *p*=0.025) and dogs that used to catch rodents (OR 6.09, 95%CI 1.16-32.01, *p*=0.04) were more likely to be infected
Wang et al., 2010 [107]	Arecoline purgation of 228 owned dogs Shiqu County (China)	Multivariable logistic regression	Parasite burden in dogs was related to the maximum burrow density of intermediate host *Ochotona spp.* (*p*=0.022)
Liccioli et el., 2012 [111]	Post-mortem examination of 61 coyotes in Calgary (Canada)	Univariable analysis	Higher prevalence in juveniles (*p*=0.035)
Catalano et al., 2012	Post-mortem	Univariable analysis	Higher infection in

[112]	examination of 91 coyotes in Alberta (Canada)		male than female coyotes (p=0.05)
Bruzinskaite-Schmidhalter et al., 2012 [83]	Post mortem examination of 310 red foxes in Lithuania	Multivariable logistic regression	Higher parasite abundance in raccoon dogs in autumn than winter (RR 0.002, 95%CI 0.0005-0.01)

Measures of association reported when available
Abbreviations: OR, odds ratio; RR, risk ratio; CI, confidence interval
