# Supplementary material for: A Systematic Review of the Epidemiology of Echinococcosis in Domestic and Wild Animals
Source: PLoS Negl Trop Dis. 2013 Jun 6;7(6):e2249. doi: 10.1371/journal.pntd.0002249 (PMC3674998; doi:10.1371/journal.pntd.0002249)
Supplement: Table S12 — Associative studies on E. multilocularis infection in intermediate hosts. (PDF) [file pntd.0002249.s013.pdf]

Table S12. Associative studies on *E. multilocularis* infection in intermediate hosts

Reference	Study Information	Statistical Method	Significant Factor
Leiby et al., 1974 [115]	Parasitological examination of 5,638 *Peromyscus maniculatus* in North Dakota (EEUU)	Analysis of variance	Age (p=0.0001), habitat (p=0.0002), season (p=0.002), age*season (p=0.003) and habitat*season (p=0.04)
Gottstein et al., 2001 [114]	Parasitological examination of 513 rodents in Fribourg (Switzerland)	Univariable analysis	Yearly fluctuation of prevalence (p<0.005) for *Arvicola terrestris*
Henttonen et al., 2001 [119]	Parasitological examination of 224 *Microtus rossiaemeridionalis* in Svalbard (Norway)	Multivariable logistic regression	Overwintered adults (p<0.001) and prevalence variation related with body weight and length (p<0.001)
Stieger et al., 2002 [102]	Parasitological examination of 1,155 rodents in Zurich (Switzerland)	Univariable analysis	Adults (p<0.001) showed higher prevalence and prevalence variation by trapping site (p=0.019) for *A. terrestris*
Hanosset et al., 2008 [93]	Parasitological examination of 1,249 rodents in Wallonia (Belgium)	Univariable analysis	Adult muskrats *Ondatra zibethicus* (p=6.56x10^{-6}) presented higher prevalence
Reperant et al., 2009 [117]	Parasitological examination of 658 rodents in Geneva (Switzerland)	Multivariable logistic regression	Body weight and geographical area (p<0.0001) for *Arvicola terrestris*
Stien et al., 2010 [118]	Parasitological examination of 387 sibling voles in Svalbard (Norway)	Multivariable logistic regression	Sample site and vole length (p<0.0001), year of sampling, sample site * year and sample site vole length (p=0.02)
Burlet et al., 2011 [116]	Parasitological examination of 856 *A. terrestris* in Zurich (Switzerland)	Multivariable logistic regression	Age (>7 months), period, area and mean day temperature included in the best-fitting model with the lowest AICc (-244.04)

Measures of association reported when available
(*) Interaction term.

Abbreviations: OR, odds ratio; CI, confidence interval; AICc, Akaike's information criterion corrected for small samples sizes.
